# Trends and Determinants of Underweight among Under-Five Children in Ethiopia: Data from EDHS

**DOI:** 10.1155/2020/3291654

**Published:** 2020-06-13

**Authors:** Haile Mekonnen Fenta, Lijalem Melie Tesfaw, Muluwerk Ayele Derebe

**Affiliations:** Department of Statistics, Bahir Dar University, Bahir Dar, Ethiopia

## Abstract

**Background and Aims:**

Malnutrition among under-five children is one of the major causes of death in low-income countries. Accordingly, health sectors in developing countries are providing renewed attention to the status of children's nutrition. This study sought to explore the trends and identify the determinants of underweight Ethiopian under-five children across time.

**Methods:**

The data in the study was obtained from the 2000, 2005, 2011, and 2016 Ethiopian Demographic and Health Surveys (EDHS). The study involved 27564 under-five children across Ethiopian regions. The status of underweight is determined using weight for age. This anthropometric index has been categorized as “underweight” (*Z*-score less than -2) and “not underweight” (*Z*-score greater or equal to -2). Logistic regression was used for each survey to detect significant determinants of underweight, while multivariate decomposition was used to determine the trends and identified the child, maternal, and household characteristics that are associated with underweight.

**Result:**

The survey in 2000, 2005, 2011, and 2016 showed that 41, 33, 29, and 24% of sampled under-five children were underweight, respectively, and after adjusting for confounders, children were more likely to be underweight if they were male (OR = 1.16, 95%CI = 1.02, 1.33) in 2016 EDHS. Children whose mother's age is below 20 (OR = 5.75, 95%CI = 1.44, 23.1)) were more likely to be underweight compared with children whose mother's age is above 45. Children whose mothers had no education and primary education only (OR = 1.65, 95% CI 1.05, 2.59 and OR = 1.43, 95% CI 1.15, 1.78, respectively) were more likely underweight compared to children whose mothers had higher education.

**Conclusion:**

Children's age, birth weight, mother's education status, and children's gender were the most common significant factors of underweight. The prevalence of underweight among under-five children declined over time which leads to an achievement in terms of meeting millennium development goals and nutritional targets. Government and concerned stakeholders should work to maintain this achievement for further reduction of underweight among under-five children.

## 1. Introduction

Malnutrition among under-five children is a major global public health problem affecting [1–3] morbidity and mortality and causing 45% of all deaths among children aged 0-59 months [4]. Studies indicated that poor child nutrition hinders children's physical and mental development and perpetuates intergenerational malnutrition that cross into adulthood. Globally, more than 178 million under-five children suffer from malnutrition [5, 6], and at any given moment, 20 million suffer from severe malnutrition. About 24.7, 7.8, and 15.1% of under-five children, respectively, were stunted (height-for-age), wasted (weight-for-height), and underweight (weight-for-age) [4].

Childhood malnutrition is most prevalent in developing countries [7]. It is the leading responsible cause of underweight. Underweight was defined as the standardized weight of age ratio less than 2. In Africa and Southeast Asia, the prevalence of chronic malnutrition and underweight was about 39.9% and 26.6%, respectively. Globally, an estimated 101 million (16%) children under five years of age were underweight in 2011 and a 36% decrease from the estimated 159 million in 1990 (52%) (WHO and UNICE report 2016 on nutritional status). Data from the previous four Ethiopian Demographic and Health Surveys (2000, 2005, 2011, and 2016) showed that the prevalence of underweight decreased over the past 16 years (41, 33, 29, and 24%, respectively), but it is still too large and we need to know the important factors affecting this status (EDHS report) [2, 3].

A community-based cross-sectional study among under-five children in western Nepal indicated that underweight in these children declined from 43% in 2001 to 27% in 2016. The study further indicated that compared to children aged less than 24, children older than 24 months were more likely to be underweight. Besides, determinants such as drinking water purification practices, growth, age of children, mother's perception of family size at birth, and growth monitoring are significantly associated with childhood underweight [8]. In a study [9], it was reported that maternal age (>35), rural residence, and prenatal follow-up are significantly associated with underweight. Lower socioeconomic status was also an important risk factor of under-five children to be underweight [10]. In 2014, 52.9% and 7% of Indian under-five children were underweight and malnourished, respectively. The prevalence of underweight among these children declined from 65% in 1980 to 33% in 2015. The reduction marked an achievement in terms of meeting millennium development and nutrition goals [11].

A cross-sectional study that involved children aged 0-24 months in West Ethiopia revealed that the prevalence of underweight among children under 0-12 months and 0-24 months was 15.1% and 8.9%, respectively [12]. The study also shows that age of child, frequency of breastfeeding, and birth weight are significant determinant factors of underweight. Moreover, in Northeast Ethiopia, the prevalence of underweight among under-five children was 24.8%. Mother's education, sex of the child, and immunization status of the child were noticed as determinants that are significantly associated with underweight [13]. On the other hand, a study conducted on underweight among children 6-59 months of age in Takusa district, Northwest Ethiopia, found 19.5% overall prevalence of underweight in the district. Of the numerous determinants considered in the study, antenatal follow-up during pregnancy and rural residence were significantly associated with underweight [9].

Though several studies [14–17] have been done on the determinants of underweight among under-five children through different statistical models and techniques, little attention is given for a comprehensive study that examine the trends of the underweight, its change over time, and determinant factors associated with underweight so far in Ethiopia. This study, therefore, is aimed at identifying the determinants of underweight and describing its trends among under-five Ethiopian children. This aim of the study is bridged by describing underweight trends among Ethiopian children and examines the pattern change of its effects using data from the four (2000, 2005, 2011, and 2016) EDHS. To our knowledge, none of the previous studies in Ethiopia examined the extent to which each selected variable contributed to the observed reduction in underweight between four specific surveys. As a result, the findings of this study will benefit policymakers at governmental and private levels in order to evaluate the progress of underweight so far and to provide an accurate policy for children aged under five.

## 2. Methods

### 2.1. Data

This study used data from national, population-based, cross-sectional survey EDHS 2000, 2005, 2011, and 2016, a worldwide project funded by the United States Agency for International Development to collect nationally representative demographic and health data on women and young children. The DHS utilized a multistage stratified cluster sampling in which sample households are selected within clusters (enumeration areas). In the selected households, measurements of height and weight were taken from children 0-59 months, women of 15-49 years, and men of 15-59 years [18].

A total of 27564 under-five children were screened for their weight-to-age measurement. Of these, 8590 were screened in 2000, 2920 in 2005, 7217 in 2011, and 8837 in 2016. EDHS granted permission to access data through the project title “Trends and Determinants of Underweight” among under-five children in Ethiopia. Nutritional status is a composite index of underweight (weight-for-age), stunting (height-for-age), and wasting (weight-for-height) [19, 20]. Therefore, this study used only one anthropometric index underweight (weight-for-age) as the main outcome variable. This anthropometric index was measured based on *Z*-score values and computed as [21]:

(1) $$\begin{array}{c} \text{Zi}=\frac{\text{AIi}-U}{\delta }, \end{array}$$

where Zi, and AIi, are the standard score (*Z*-score) and anthropometric index of child *i*, respectively. whereas *U* and *δ* are the median and standard deviation of the reference population, respectively. This anthropometric index has been categorized as “underweight” (*Z*-score less than -2) and “not underweight” (*Z*-score greater or equal to -2). In this sense, a child that belongs to the “not underweight” category might be normal weight, overweight, or obese. However, in Ethiopian context, most reports indicated extremely low proportion of overweight and obese [18].

Independent variables include household characteristics (residence, region, wealth index, source of drinking water, and toilet facilities), maternal characteristics (maternal/paternal education and occupation, mother's age, number of under-five children, and children living with mother), and child characteristics (sex, age, birth order, birth type, and childhood illness) and child caring practices (exclusive breastfeeding).

### 2.2. Statistical Analysis

The outcome variable was coded as a binary variable (“underweight” = 1 and “not underweight” (contains normal, overweight, and obese) = 0), and the data analysis was performed using STATA 14. The data analysis part includes three important stages. First, descriptive statistics and trends were analyzed in selected recoded background variables among all births in the five years preceding each survey. Second, binary logistic regression analysis was carried out to identify factors associated with underweight in children. Third, to understand the extent to which each selected covariate contributed to the observed trend in underweight prevalence, multivariate decomposition procedures were implemented. This multivariate decomposition provides a way to analyze differences in the outcome between two groups or, as in this case, between two points of time (like 2000 and 2016 survey years). The decomposition procedure divides the total decline in underweight into two portions: the portion that can be attributed to the change in composition or the prevalence of a set of indicators (referred to as the endowments portion) and the portion that can be attributed to the change in the effect of these indicators (referred to as the coefficient portion) [22]. The formula is given by

(2) $$\begin{array}{c} \Delta Y^{i-j}=\left(X^{i}-X^{j}\right)\beta^{i}+X^{j}\left(\beta^{i}-\beta^{j}\right),i\neq j, \end{array}$$

where *i*, *j* = 2000, 2005, 2010, and 2016, Δ*Y* is the difference in mean prediction of underweight between year *i* and year *j*, given that of different characteristics *X*.

*β* is the estimated regression coefficients.

(*X*^*i*^ − *X*^*j*^)*β*^*i*^ represents the difference due to endowment between the *i*^th^ and *j*^th^ years.

*X* ^*j*^(*β*^*i*^ − *β*^*j*^) represents the difference due to coefficients between the *i*^th^ and *j*^th^ years.

## 3. Results

Table 1 presents the percentage distribution of underweight in under-five children based on children's maternal and household characteristics reports from 2000-2016 EDHS. Accordingly, the analysis included data from 8590 children in the 2000, 2920 in the 2005, 7217 in the 2011, and 8837 children in the 2016 EDHS. The table showed the trends in the underweight, disaggregated by household, children, and mother level characteristics. The result indicated a long-term trend in under-five underweight children in Ethiopia, a period of nearly two decades, 2000 to 2016.

In 2000, 41% of children were underweight and this declined to 24% in 2016. This shows a persistent decline of children underweight from 2000 to 2016. However, in 2005, the proportion of households with poorer economic status was 24.7% and increased to 30.4% in 2016. Unfortunately, in 2000, the data of the household related to socioeconomic status was not recorded. Among the total study participants, the number of underweight is higher in males than in females for all survey periods (2000-2016). Majority of the children (higher than 30%) were born within the birth interval of 24 to 35 months, and the gap increased with the increase in the survey periods.

In 2000 and 2016, 92.8% and 93.3% of under-five children, respectively, were born from the rural area. Percentage of mothers with primary education increased over the survey periods, and the proportion of women with currently working status is increased from time to time.

The EDHS in 2005, 2011, and 2016 showed that the highest proportion of women had the poorest socioeconomic status. About 20% of all the households were below the middle wealth group and did not vary a great deal during the period. The percentage of households with piped drinking water decreased during the study period, from 2.8 percent in 2005 to 0.8 percent in 2016 (Table 1).

Table 2 depicts the adjusted odds ratio (AOR) for all the factors that satisfied the univariate analysis (crude odds ratio) criteria in the model. It shows that in all four surveys, the risk of underweight was higher in under-five male children rather than in females. Specifically, in the 2011 and 2016 EDHS datasets, males were more likely to be underweight (AOR = 1.22, 95% CI: 1.05, 1.40 and AOR = 1.16, 95% CI: 1.02, 1.33, respectively). The birth interval (in months) of children was statistically significant only in 2000 EDHS dataset. Birth type was statistically affected by the underweight in children. In 2016 EDHS, children from mothers with singleton births were approximately 0.29 (AOR = 0.29, 95% CI:0.16, 0.53) times less likely to be underweight as compared to children from mothers who had multiple births.

The multivariable analysis showed that in the last two EDHS data, male rather than female children were more likely to be underweight (OR = 1.22, 95%CI = 1.05, 1.40 and OR = 1.16, 95%CI = 1.02, 1.33, respectively). Similarly, according to the 2016 EDHS, children born from a household of poor socioeconomic status were more likely to be underweight rather than under-five children from a family with rich socioeconomic status (OR = 1.65, 95%CI = 1.20, 2.26). More generally, in 2016, children's gender, mothers' age, and educational status and birth types have significant association with underweight in children. In all the four surveys children's age was significantly associated with underweight, and the odds of having overweight increased with increase in the age of children.

The multivariate decomposition of the analysis (see Table 3) identified factors that are associated with the reduction of underweight among under-five children in different survey years of Ethiopia. The underweight among under-five children across the surveys has two parts: one representing changes in the distribution of household, child, and mother characteristics, also called endowments, and the other representing the size of the effect of the characteristics (coefficients). Characteristic differences did not have a significant effect on the underweight of under-five children between 2011 and 2016 EDHS. The results in the endowment column quantifying the difference between odds of the underweight among under-five children for each characteristic between the two points in time by assuming the other characteristics are constant.

The underweight among under-five children declined by 19.5 points between the surveys of 2000 and 2016. Moreover, the underweight among under-five children declined by 9.95 and 5.01 between 2005 and 2016 and 2011 and 2016 EDHS, respectively.

## 4. Discussion

Childhood malnutrition is a major problem in developing nations including Ethiopia. In the present study, we looked at the effects of socioeconomic factors on childhood underweight and its trend in Ethiopia using data from four cross-sectional EDHS datasets. First, the association of each child's maternal and household characteristics with underweight was identified through logistic regression. Second, the maternal and household characteristic's coefficient difference among 2000, 2005, 2011, and 2016 EDHS was determined using multivariate decomposition analysis.

In the country, for the last two decades, it is estimated that approximately 41% in 2000 and 24% in 2016 of children were underweight, respectively; this promising result may be due to the great effects of the government of the country and different stakeholders to create an awareness of the nutritional status of mothers and their child feeding practices, improvements of sanitations, and the like. The improvement of sanitation, feeding practice improvements and education of parents have an impact to minimize the child underweight status and this result is consistent with the study conducted in different countries [23, 24]. The findings of this study showed that children raised in rural areas have a better decline of underweight than urban children; this might be the fact that the commitment of the government of the country is increasing awareness to the rural community-related child health and feeding practices. This result is consistent with the study conducted in India, Vietnam, and other countries [23–26]. The four EDHS data showed that underweight is decreased over time and the result of the study is in line with a study in [11], which reported that the prevalence of underweight on under-five children declined from 65% in 1980 to 33% in 2015.This reduction in underweight is a good indicator in terms of achieving millennium development goals and nutritional targets. In all the four (2000, 2005, 2011, and 2016) survey periods, males rather than females were more underweight among the under-five children. In 2016, children's gender, mother's age, educational status, and types of birth have a significant association with underweight status of under-five children. This is consistent with studies in [8, 9] in which both reported mother's and children's ages to be significantly associated with underweight in children.

It was also found that under-five children from poor economic status households were more vulnerable to underweight (OR = 1.65, CI = (1.20, 226)) which is in line with [10]. Moreover, lower socioeconomic status is an important risk factor of underweight under-five children as it leads to poor childhood nutrition during and hinder physical and mental development of children triggering vicious cycle of intergenerational malnutrition [4, 20]. Children's age has a significant effect on children underweight as underweight increase with increase in age. This is consistent with findings in [8] which reported that compared to children younger than 24 months, children who were older than 24 months were more likely to be underweight.

In the 2005 EDHS of this study, breastfeeding had a significant association with underweight that is in line with a study in West Ethiopia [12] and in contrast with findings of this study reported in 2000, 2011, and 2016 EDHS. The prevalence of underweight in Northeast Ethiopia [13], 24.8%, is greater by 0.8% compared to the prevalence of underweight in Ethiopia in 2016 EDHS. However, the reverse is true in a study among under-five children in Northwest Ethiopia [9] indicating that the prevalence of underweight was 19.5% which is quite different and lower in proportion compared to the prevalence of underweight of under-five children in this study from 2016 EDHS, 24%. The change in the distribution of underweight in terms of place of residence, access to improve water source, birth order, types of birth, and educational status were significantly different between 2000 and 2016 EDHS.

This research study has strength over other cross-sectional studies that involved children at a single point of time because it is incorporating children from four (2000, 2005, 2011, and 2016) EDHS consecutive periods.

### 4.1. Limitation of the Study

According to DHS, data is collected once per five years. Thus, the data of underweight in this study are considered only once per five years, in 2000, 2005, 2011, and 2016, four successive surveys. Hence, it is doubtful if there are lots of up and down trends within the five-year interval. Moreover, this study involved only children aged under five.

## 5. Conclusions

Children's age, birth weight, mother's education status, and children gender were the most common significant factors of underweight. The prevalence of underweight in under-five children declined from 41% in 2000 to 24% in 2016, and the effect of child, mother, and household characteristics differed significantly overtime. Thus, over the last two decades, many developing countries including Ethiopia have made remarkable progress in reducing underweight in under-five children. This reduction leads to an achievement in terms of meeting nutrition targets and millennium development goals which were used as an input for policymakers to update their strategy and designing strategies to improve the nutritional status of children in Ethiopia. The government and concerned stakeholders should work to maintain this achievement for further reduction of underweights among under-five children.

## Figures and Tables

**Table 1:** Percent distribution of under-five children, maternal and household characteristics: Ethiopian Demographic and Health Survey of 2000, 2005, 2011, and 2016.

Characteristics	2000 (*n* = 8590)	2005 (*n* = 2920)	2011 (*n* = 7217)	2016 (*n* = 8837)
Prevalence (%)	41	33	29	24
Children gender(%)				
Female	48.6	48.6	45.6	45.9
Birth interval in months				
First birth	14.9	13.6	17.4	16.2
7-17	6.4	6.4	7.4	9.8
18-23	9.5	11.9	9.9	10.3
24-35	32.7	33.3	31.3	30.1
36-47	20.9	20.8	18.8	16.5
48-59	9.3	8.1	8.2	8.2
60+	6.3	5.9	7.0	9.0
Breastfeeding (yes as ref.)				
No	76.4	79.6	73.7	70
Had diarrhea recently (yes as ref.)				
No	29	22	17	15.2
Place of residence (%)				
Rural	92.8	95.5	92.8	93.3
Region (%)				
Amhara	28.7	27.1	25.6	24.2
Afar	1	0.9	1.4	1.4
Tigray	7	7.8	8.3	6.6
Oromia	37.2	36	38.9	41.5
Benishangul-Gumuz	0.9	1.2	1.3	1.6
Dire-Dawa	0.3	0.3	0.3	0.5
SNNPR	23.4	20.5	20.4	18.2
Harari	0.1	0.2	0.2	0.3
Somalia	0.7	5.4	3	5
Gambella	0.2	0.2	0.2	0.2
Addis ababa	0.5	0.4	0.4	0.5
Mother's age (year)				
<20	3.1	3.6	3.7	3.3
20-24	19	18.4	18.2	18.4
25-29	26	26.8	32.3	29.3
30-34	22.5	22	21.5	23.8
35-39	17	18.5	14.1	16.2
40-44	8.9	7.3	8.2	6.8
45-49	3.5	3.2	2	2.2
Mother's education status/mother				
No education	86	84	75.1	75.8
Primary	11	14.5	23.8	20.9
Secondary	2.9	1.49	0.9	2.3
Higher	0.1	0.01	0.2	1
Mother currently working				
No	58	25.8	32.8	26.1
Socioeconomic status (SES)				
Poorest		24.7	28.1	30.4
Poorer		25.3	25.3	26.8
Middle		20.8	21	20.6
Richer		17.9	17.8	13.3
Richest		11.3	7.7	8.9
Sources of drinking water				
Piped	2.8	1.5	0.9	0.8
Borehole/well	87.5	16.3	14.9	24.3
Surface/rain/pond/lake/tank	0.01	30.6	59.3	50.7
Others	11.49	52.2	24.3	22.2
Type of toilet facility				
Flush	0.1	1.6	1.1	0.8
Pit latrine	11.4	25.4	42.9	51.7
No facility	88.5	73	56	47.5
Husband/partner's education level				
No education	70.1	62.3	57.9	57.8
Primary	22.5	29.7	39.19	35.2
Secondary	6.8	6.6	2.9	5.3
Higher	0.6	12.4	0.01	1.7
BMI of mothers (%)				
Normal (18-5-24.9)	12.6	10.7	12.2	1
Too thin for their height (<18.5),	6.3	6.5	7.6	8.73
Overweight (25-29.9)	80	80.3	77.9	79.7
Obese (>+30))	1	2.5	2.3	11

**Table 2:** Parameter estimates of household, maternal, and child characteristics: EDHS of 2000, 2005, 2011, and 2016.

	Adjusted OR (95% CI)			
Characteristics	2000 (*n* = 8590)	2005 (*n* = 3873)	2011 (*n* = 9611)	2016 (*n* = 8837)
Children age (in year) (5 ref.)				
4	0.23 (0.18, 0.30)^∗^	0.17 (0.11, 0.27)^∗^	0.44 (0.32, 0.61)^∗^	0.52 (0.41, 0.65)^∗^
3	0.22 (0.17, 0.28)^∗^	0.17 (0.12, 0.26)^∗^	0.33 (0.25, 0.45)^∗^	0.45 (0.35,0.58)^∗^
2	0.26 (0.20, 0.34)^∗^	0.16 (0.11, 0.26)^∗^	0.32 (0.24, 0.45)^∗^	0.45 (0.36, 0.57)^∗^
1	0.25 (0.19, 0.33)^∗^	0.15 (0.10, 0.25)^∗^	0.32 (0.24, 0.45)^∗^	0.38 (0.30, 0.49)^∗^
Children gender (female as ref.)				
Male	1.13 (0.97, 1.29)	1.10 (0.91, 1.32)	1.22 (1.05, 1.40)^∗^	1.16 (1.02, 1.33)^∗^
Birth interval in months (60+ as ref.)				
First	0.71 (0.51, 0.98)^∗^	0.80 (0.51, 1.26)	0.81 (0.57, 1.13)	0.68 (0.36, 1.29)
7-17	0.71 (0.51, 0.99)^∗^	0.68 (0.44, 1.27)	0.96 (0.70, 1.31)	0.74 (0.43, 1.27)
18-23	0.75 (0.59, 0.95)	0.89 (0.60, 1.33)	0.94(0.73, 1.22)	0.83 (0.53, 1.30)
24-35	0.78 (0.60, 1.01)	0.93 (0.62, 1.40)	0.93(0.70, 1.23)	1.05 (0.62, 1.77)
36-47	0.91 (0.67, 1.24)	0.81 (0.50, 1.31)	1.17(0.81, 1.69)	1.17 (0.67, 2.06)
48-59	1.05 (0.75, 1.48)	0.99 (56, 1.78)	1.21(0.80, 1.84)	1.27 (0.70, 2.28)
Place of residence (rural as ref.)				
Urban	0.91 (0.60, 1.36)	0.84 (0.49, 1.43)	1.11 (0.63, 1.97)	0.97 (0.65, 1.47)
Caregiver's age (*y*) (mother's age < 20 as ref.)				
20-24	0.99 (0.68, 1.47)	0.78 (0.45, 1.37)	1.28 (0.80, 2.03)	2.18 (1.11, 4.32)^∗^
25-29	0.80 (0.5, 1.33)	0.89 (0.53, 1.52)	1.19 (0.71, 2.01)	2.13 (1.03, 4.42)^∗^
30-34	0.74 (0.48, 1.4)	0.78 (0.46, 1.32)	1.21 (0.71, 2.06)	2.19 (1.06, 4.52)^∗^
35-39	0.83 (0.52, 1.31)	0.61 (0.34, 1.01)	1.56 (0.93, 2.64)	1.66 (0.80, 3.44)
40-44	0.87 (0.54, 1.42)	1.19 (0.64, 2.21)	0.91 (0.53, 1.58)	1.36 (0.59, 3.16)
45-49	1.11 (0.63, 1.92)	0.96 (0.45, 2.02)	1.79 (0.85, 3.76)	5.75 (1.44, 23.1)^∗^
Mother's education status (higher ref.)				
Secondary	1.25 (0.98, 1.61)	1.34 (0.98, 1.86)	0.95 (0.76, 1.20)	1.43 (1.15, 1.78)^∗^
Primary	1.67 (1.04, 2.67)	1.88 (0.85, 4.18)	1.21 (0.46, 3.20)	1.65 (1.05, 2.59)^∗^
No education	2.63 (0.98, 3.21)	2.45 (0.28, 21.18)	1.75 (0.33, 9.27)	1.54 (0.68, 3.46)
Socio economic status (SES) (richest as ref.)				
Richer		0.90 (0.68, 1.12)	0.94 (0.76, 1.18)	1.20 (0.86, 1.41)
Middle		1.18 (0.87, 1.58)	1.2 (0.91, 1.56)	1.29 (0.96, 1.74)
Poor		1.29 (0.92, 1.82)	1.3 (0.93, 1.82)	1.65 (1.20, 2.26)^∗^
Poorest		1.35 (0.89, 2.05)	2.03 (1.15, 3.59)^∗^	1.39 (0.89, 2.17)
Breastfeeding (no as ref.)				
Yes	0.86 (0.69, 1.08)	0.58 (0.44, 0.77)^∗^	0.72 (0.27, 0.99)	0.86 (0.57, 1.31)
Had diarrhea recently (no as ref.)				
Yes	0.61 (0.51, 0.73)^∗^	0.75 (0.56, 0.99)	0.70 (0.57, 0.85)^∗^	0.62 (0.48, 0.82)∗
Marital status (not married as ref.)				
Married	0.79 (0.78, 1.09)	0.97 (0.58, 1.62)	0.76 (0.61, 0.96)^∗^	2.50 (0.73, 8.60)
Sex of household (male as ref.)				
Female	0.72 (0.56, 0.93)	0.91 (0.64, 1.30)	0.83 (0.68, 1.08)	0.97 (0.76, 1.24)
Vaccination (no. as ref.)				
Yes	0.77 (0.64, 0.94)	1.09 (0.86, 1.38)	0.86 (0.68, 1.08)	0.56 (0.39, 0.82)
Sources of drinking water (piped as ref.)				
Borehole or well	1.02 (0.47, 2.22)	1.14 (0.37, 3.56)	0.74 (0.36, 1.50)	1.33 (0.47, 3.72)
Surface/rain/pond/Lake/tank	1.01 (0.42, 2.43)	1.08 (0.34, 3.46)	0.62 (0.31, 1.21)	1.50 (0.53, 4.05)
Others	0.99 (0.50, 2.00)	0.89 (0.30, 2.71)	0.56 (0.30, 1.03)	1.13 (0.41, 3.13)
Type of toilet facility (flush as ref.)				
Pit latrine	1.53 (0.29, 8.04)	1.43 (0.47, 4.32)	0.67 (0.33, 1.32)	0.62 (0.36, 1.10)
No facility	1.56 (0.31, 7.89)	1.29 (0.44, 3.85)	0.50 (0.24, 1.07)	0.55 (0.31, 0.97)^∗^
Currently pregnant (no as ref)				
Yes	1.01 (0.74, 1.38)	0.78 (0.53, 1.13)	0.80 (0.57, 1.10)	0.79 (0.46, 1.36)
Size of a child (normal as ref.)				
Small	0.67 (0.57, 0.78)^∗^	0.67 (0.52, 0.85)^∗^	0.67 (0.54, 0.82)^∗^	0.58 (0.42, 0.80)^∗^
Large	1.17 (0.99, 1.38)	1.21 (0.92, 1.58)	1.44 (1.20, 1.73)	1.07 (0.77, 1.48)
Respondent currently working (no as ref.)				
Yes	0.96 (0.82, 1.12)	0.79 (0.61, 1.01)	1.045 (0.83, 1.32)	0.94 (0.79, 1.13)
Husband's education level (higher as ref.)				
Secondary	1.33 (1.11, 1.60)^∗^	1.02 (0.78, 1.33)	1.14 (0.94, 1.37)	1.18 (0.98, 1.42)
Primary	1.51 (1.11, 2.04)^∗^	1.03 (0.68, 1.54)	1.44 (0.64, 3.26)	1.16 (0.76, 1.77)
No education	2.26 (0.81, 6.29)	0.69 (0.18, 2.65)	1.95 (0.45, 8.41)	1.69 (1.12, 2.56)^∗^
Types of birth (multiple as ref.)				
Singleton	0.68(0.38,1.20)	0.34(0.12,1.01)	0.42(0.22,0.81)^∗^	0.29 (0.16, 0.53)^∗^
BMI of mothers (normal (18-5-24.9)				
Under weight (<18.5) ref.)	1.17 (0.78, 1.75)	0.78 (0.47, 1.31)	0..91 (0.62, 1.34)	0.20 (0.07, 0.58)
Overweight (25-29.9)	1.72 (1.39, 2.14)	2.04 (1.44, 2.89)	1.75 (1.37, 2.25)	0.86 (0.17, 4.32)
Obese (>+30))	4.11 (2.13, 7.94)	2.94 (1.52, 5.67)	2.73 (1.61, 4.63)	1.37 (0.49, 3.85)
Current use of contraceptive (no as ref.)				
Yes	1.30 (0.97, 1.09)	1.03 (0.74, 1.42)	0.99 (0.80, 1.23)	1.05 (0.88, 1.27)
Constant	5.44 (1.03, 28.71)	6.28 (1.18, 33.40)	19.43 (4.93, 76.65)	7.24 (0.75, 69.15)

Key: ^∗^significant at 0.05 significance level; ref.: reference category.

**Table 3:** Multivariate decomposition of different characteristics from 2000 to 2016 of EDHS.

	2000 EDHS-2016 EDHS		2005EDHS-2016 EDHS		2011EDHS-2016 EDHS	
Characteristics	Endowments	Coefficients	Endowments	Coefficients	Endowments	Coefficients
Household level						
Place of residence	1.5*E*-2^∗∗^	0.123^∗^	1.9*E*-4	0.103	3.02*E*-4	0.050
Access to improved water source	-2.2*E*-3^∗^	-0.008	3.9*E*-3	-0.011	2.51*E*-4	0.002
Access to improved toilet source	0.022^∗∗^	-0.040	2.8*E*-3	-0.021	1.1*E*-3	-0.005
Wealth index			-8.2*E*-3^∗∗^	0.042	1.3*E*-3^∗^	0.019
Child level						
Sex of child	-15*E*-4	0.046^∗^	-4.6*E*-4	0.010	3.9*E*-5	-0.018
Anemia level			-3.4*E*-2^∗∗^	0.032	-4.3*E*-3^∗∗^	-0.322
Diarrhea status			1.6*E*-3^∗^	8.9*E*-4	7.7*E*-4	0.003
Currently breastfeeding	5.0*E*-5	0.005	1.3*E*-3	0.017	3.4*E*-4	-7.3*E*-4
Birth order	1.4*E*-3^∗^	0.022	2.1*E*-3^∗^	0.074	2.1*E*-3^∗∗^	0.013
Types of birth	-1.4*E*-3^∗∗^	-1.6*E*-4	-1.2*E*-3^∗^	0.004^∗^	-1.4*E*-4	1.9*E*4
Birth interval	-7.6*E*-5	0.030	-1.1*E*-3	0.013	-3.9*E*-4	0.011
Mother level						
Educational status	0.017^∗∗∗^	0.006	8.1*E*-3^∗∗^	-0.001	7.4*E*-3^∗∗^	8.6*E*-3
Religion	-3.8*E*-4	-0.005	-1*E*-4	-0.011	2.8*E*-5	-2.7*E*-4
Working status	5.4*E*-3	-0.002	-7.1*E*-4	-0.003	1.6*E*-4	-0.009
Occupation			-7*E*-5	-0.009	1.2*E*-4	0.026
Age category		0.065	1.8*E*-6	-0.024	1.2*E*-4	-0.027
Total children ever born	-0.027	-0.028	-1.5*E*-4	-0.009	-1.5*E*-3	-0.026
Births in last five years	-0.039	-0.040	-1.1*E*-4^∗^	-0.029	-6.5*E*-3^∗^	-0.038
Age at first birth	0.047	0.0460	1.4*E*-3^∗^	0.043	1.2*E*-3^∗^	0.066
Constant		-0.070		-0.103		0.0065
Total percent	3.63*E*-2				2.4E-3	
Difference (%)	-19.53		-9.97		-5.01	

Key: ^∗^*p* value < 0.05; ^∗∗^*p* value < 0.01; ^∗∗^*p* value < 0.001.

## Data Availability

The data can be accessed from the DHS site by providing a formal request of research proposal.
